# Comprehensive Analysis of GLUT1 Immune Infiltrates and ceRNA Network in Human Esophageal Carcinoma

**DOI:** 10.3389/fonc.2021.665388

**Published:** 2021-05-28

**Authors:** Xu-Sheng Liu, Yan Gao, Li-Bing Wu, Hua-Bing Wan, Peng Yan, Yang Jin, Shi-Bo Guo, Ya-Lan Wang, Xue-Qin Chen, Lu-Meng Zhou, Jian-Wei Yang, Xue-Yan Kui, Xiao-Yu Liu, Zhi-Jun Pei

**Affiliations:** ^1^ Department of Nuclear Medicine and Institute of Anesthesiology and Pain, Taihe Hospital, Hubei University of Medicine, Shiyan, China; ^2^ Hubei University of Medicine, Shiyan, China; ^3^ Hubei Key Laboratory of Embryonic Stem Cell Research, Shiyan, China

**Keywords:** GLUT1, esophageal carcinoma, immune infiltration, m6A modification, ceRNA

## Abstract

**Background:**

Glucose transporter 1 (GLUT1) is encoded by the solute carrier family 2A1 (SLC2A1) gene and is one of the glucose transporters with the greatest affinity for glucose. Abnormal expression of GLUT1 is associated with a variety of cancers. However, the biological role of GLUT1 in esophageal carcinoma (ESCA) remains to be determined.

**Methods:**

We analyzed the expression of GLUT1 in pan-cancer and ESCA as well as clinicopathological analysis through multiple databases. Use R and STRING to perform GO/KEGG function enrichment and PPI analysis for GLUT1 co-expression. TIMER and CIBERSORT were used to analyze the relationship between GLUT1 expression and immune infiltration in ESCA. The TCGA ESCA cohort was used to analyze the relationship between GLUT1 expression and m6A modification in ESCA, and to construct a regulatory network in line with the ceRNA hypothesis.

**Results:**

GLUT1 is highly expressed in a variety of tumors including ESCA, and is closely related to histological types and histological grade. GO/KEGG functional enrichment analysis revealed that GLUT1 is closely related to structural constituent of cytoskeleton, intermediate filament binding, cell-cell adheres junction, epidermis development, and P53 signaling pathway. PPI shows that GLUT1 is closely related to TP53, GIPC1 and INS, and these three proteins all play an important role in tumor proliferation. CIBERSORT analysis showed that GLUT1 expression is related to the infiltration of multiple immune cells. When GLUT1 is highly expressed, the number of memory B cells decreases. ESCA cohort analysis found that GLUT1 expression was related to 7 m6A modifier genes. Six possible crRNA networks in ESCA were constructed by correlation analysis, and all these ceRNA networks contained GLUT1.

**Conclusion:**

GLUT1 can be used as a biomarker for the diagnosis and treatment of ESCA, and is related to tumor immune infiltration, m6A modification and ceRNA network.

## Introduction

Esophageal carcinoma (ESCA) is one of the most common malignancies of the upper digestive tract worldwide, and esophageal squamous cell carcinoma (ESCC) is the most common pathological subtype of ESCA. ESCA has the characteristics of high malignancy, poor prognosis, and high mortality, which has seriously threatened human life and health. Although some progress has been made in the diagnosis and treatment of esophageal diseases, the prognosis of patients with middle and advanced ESCA is still extremely poor, with a 5-year survival rate of only 15%-20%. The molecular mechanism of the formation and progression of ESCA is an extremely complex process involving cell cycle regulation and signal transduction, which makes effective treatment of ESCA more difficult. Therefore, further studying the pathogenesis of ESCA and providing new molecular targets for the early diagnosis and treatment of tumors has more important practical significance and theoretical value.

Glucose transporter 1 (GLUT1) is the first glucose transporter discovered and one of the glucose transporters with the greatest affinity for glucose (1). GLUT1 is encoded by the solute carrier family 2A1 (SLC2A1) gene (2), and its crystal structure was analyzed for the first time in 2014 (3). GLUT1 is widely present in most tissues of the human body, but in normal tissues and benign lesions, the expression level of GLUT1 is low, and high expression is often related to cancer, which may indicate a poor prognosis or recurrence (4). GLUT1 not only maintains the normal functions of the human body, but also plays an important role in the occurrence and development of tumors, especially in the glycolysis process of tumor cells (5). Tumor cells need to consume a lot of glucose when they grow and proliferate. However, GLUT1 is one of the key proteins that transport glucose into cells. Currently, studies have shown that GLUT1 is highly expressed in oral (4), gastric (5), breast (6), colorectal (7), and ovarian cancers (8). In previous studies (9), we found that GLUT1 was highly expressed in ESCA and strongly correlated with metabolic parameters of ^18^F-FDG PET/CT imaging, but we failed to carry out more studies on the biological function of GLUT1 in ESCA.

Tumor immunotherapy, N6-methyladenosine (m6A) and ceRNA regulatory network are the new directions of tumor gene therapy and are widely used in the study of the mechanism of ESCA. Na et al. (10) found that GLUT1 is highly expressed in lung adenocarcinoma, and the expression level is negatively correlated with immune score. ^18^F-FDG uptake in immunocompromised lung adenocarcinoma patients is positively correlated with GLUT1 expression, but negatively correlated with immune score. This result indicates that the competitive uptake of glucose by cancer cells and immune cells in the tumor microenvironment may be caused by the different expression levels of GLUT1 in the cells. Chen et al (11). found that the key gene of m6A, METTL3, induced GLUT1 translation in an m6a-dependent manner, which enhanced the glucose uptake and lactate production of colorectal cancer cells, which in turn led to the activation of mTORC1 signaling and the development of colorectal cancer. Chen et al. (12) found that the CircRNA_100290/miR-378a/GLUT1 ceRNA regulatory network plays an important role in oral squamous cell carcinoma. Overexpression of GLUT1 can rescue the reduction of tumor cell proliferation and glycolysis caused by down-regulation of circRNA. However, there is little research on the overall understanding of GLUT1 in ESCA, especially the relationship between GLUT1 and tumor immunotherapy, m6A modification and ceRNA regulatory network.

In this study, we analyzed the differences in GLUT1 expression in different cancers by analyzing The Cancer Genome Atlas (TCGA) and various public databases. Using multi-dimensional analysis to evaluate the gene and functional network related to the expression of GLUT1 in ESCA, and to explore the relationship between its expression differences and tumor immunity, m6A modification, and ceRNA regulatory network, provide a theoretical basis for discovering possible molecular pathways.

## Materials and Methods

### Ethics Statement

This study proposal has been approved by the Ethics Committee of Taihe Hospital Affiliated of Hubei University of Medicine (Shiyan, China) and conducted in accordance with the research principles described in the Helsinki Declaration.

### Oncomine Analysis

Oncomine (www.oncomine.org) is a publicly accessible database of oncogene chip information used to analyze the transcription level of GLUT1 in various cancers (13). This database uses Student’s t test to compare the transcription levels of GLUT1 in normal controls and clinical cancer specimens. In this study, the fold change > 2 and cut-off of P-value < 0.0001.

### TIMER Analysis

Tumor immune to assess resource (TIMER, www.cistrome.shinyapps.io) is a reliable and convenient database, including gene expression profiles from the TCGA database. The TIMER tool can be used to estimate immune cell infiltration and evaluate its clinical impact (14, 15).

In this study, we used TIMER to evaluate the transcription level of GLUT1 in a variety of tumors and analyzed the correlation between GLUT1 transcription level and immune cell infiltration, including B cells, neutrophils, CD4 + T cells, macrophages, CD8 + T cells, and dendritic cells, as well as the tumor purity. We used the TIMER tool to analyze the correlation between GLUT1 and immune cell markers to evaluate the role of GLUT1 in tumor immunity. Immune cell gene markers are selected from the website of R&D Systems (www.rndsystems.com/cn/resources/cell-markers/immune-cells). These gene markers include markers of B cells, CD8 + T cells, follicular helper T cells (Tfh), T-helper 1 (Th1) cells, T-helper 2 (Th2) cells, T-helper 17 (Th17) cells, Treg, T cells exhausted, macrophages, M1 macrophages, M2 macrophages, tumor-associated macrophages (TAM), monocytes, natural killer (NK) cells, neutrophils, and dendritic cells (DC). In addition, we used the somatic copy number alteration (SCNA) module of the TIMER tool to link the genetic copy number variations (CNV) of GLUT1 with the relative abundance of tumor infiltrating cells.

### TCGA Data

TCGA (www.tcga-data.nci.nih.gov/tcga/) contains more than 10,000 samples of 39 tumor types (16). We downloaded ESCA RNA-seq data from the Genomic Data Commons (GDC, https://portal.gdc.cancer.gov/) database, which included 162 tumor samples and 11 normal samples. In this study, we used TCGA-ESCA data to analyze the expression of GLUT1 and the correlation between GLUT1 expression and clinicopathological characteristics. In addition, we also analyzed the correlation between the expression level of GLUT1 and the expression of m6A-related genes in ESCA samples and the differences expression in m6A-related gene between the high and low GLUT1 expression groups. m6A related genes include METTL3, YTHDC1, YTHDC2, METTL14, RBM15, RBM15B, IGF2BP1, IGF2BP2, IGF2BP3, VIRMA, WTAP, YTHDF1, YTHDF2, YTHDF3, ZC3H13, HNRNPA2B1, HNRNPC, RBMX, FTO and ALKBH5 (17).

### GEO Data

We downloaded the RNA sequencing data of ESCA from the Gene Expression Omnibus (GEO, www.ncbi.nlm.nih.gov/geo) database to analyze the transcription level of GLUT1. (GSE38129, n=60; GSE23400, n=106).

### Cell Lines and Cell Culture Reagents

Human ESCA cell lines ECA109 and KYSE-150 and normal human squamous esophageal cell line Het-1A were obtained from the American Type Culture Collection (Manassas, VA, USA). The cells were maintained in DMEM high glucose medium (Hyclone, Logan, UT, USA) supplemented with 10% FBS (Gibco, USA) and 1% antibiotics (penicillin-streptomycin, Gibco, USA).

### RNA Extraction and qRT-PCR

The implementation method refers to previous study (18). Total RNA was isolated from cells using Trizol reagent (Invitrogen, Carlsbad, CA, USA). Use Prime Script RT reagent kit (Takara, Dalian, China) for reverse transcription, and then use SYBR Prime Script RT PCR kit (Takara, Dalian, China) for qRT-PCR. Use GAPDH as an internal reference and use the 2^-△△Ct^ method to calculate the results. GLUT1 primer sequences: forward primer CTTTGTGGCCTTCTTTGAAGT and reverse primer CCACACAGTTGCTCCACAT. GAPDH primer sequences: forward primer GGAGCGAGATCCCTCCAAAAT and reverse primer GGCTGTTGTCATACTTCTCATGG.

### Immunohistochemistry

Clinical samples were obtained from 50 patients with ESCA who were surgically treated at Taihe Hospital Affiliated of Hubei University of Medicine from February 2016 to September 2017. The content of GLUT1 was detected by IHC according to the method previously described (9). The ESCA tissue and the paracarcinoma tissues were prepared into 3 μm paraffin sections and incubated with mouse monoclonal antibodies of GLUT1 (1:200, Abcam, USA) at 4°C overnight in a refrigerator. The sections were coupled with the goat anti-mouse IgG-HRP secondary antibody (1:2000, Abcam, USA) at room temperature for 1.5 h, then each incubated section was stained with DAB reagent, and finally counterstained with hematoxylin.

### LinkedOmics Analysis

The LinkedOmics database (http://www.linkedomics.org/login.php) is a web-based platform that can provide comprehensive multi-omics data analysis tools for the TCGA database (19). The Pearson correlation coefficient was used for statistical analysis of GLUT1 co-expression and displayed in the form of volcano map and heat map. The rank criterion was an FDR<0.05.

### R Software

The ClusterProfiler package (version: 3.18.0) of R was employed to analyze the GO function and KEGG pathway enrichment of potential targets. Use the ggplot2 software package to visualize the analysis data. The CIBERSORT package was used to evaluate the relative proportion of 22 immune infiltrating cells in tumor samples when GLUT1 was high or low expression.

### STRINGS Analysis

STRINGS (www.string-db.org) is an online analysis website that contains all publicly available protein-protein interaction (PPI) data. In this study (20), we used STRING to perform PPI network analysis on GLUT1.

### Prediction of miRNA

Use starBase3.0 (www.starbase.sysu.edu.cn) online website to predict the target miRNA of GLUT1, and the prediction results include the analysis of PITA, miRanda and TargetScan (21). In addition, we analyze the correlation between target miRNA expression and GLUT1 expression to screen for miRNAs that are more in line with ceRNA conditions. Finally, use TargetScan (http://www.targetscan.org/vert_72) online tool to predict the potential binding site of target miRNA and GLUT1 (22).

### Prediction of lncRNA and ceRNA Network Construction

Use miRNet2.0 (www.mirnet.ca/miRNet/home.xhtml) (23) and starBase to predict the target lncRNA of miRNA, miRNA includes has-miR-140-5p, has-miR-148a-3p and has-miR-148b-3p. In addition, we analyze the correlation between target miRNA expression and lncRNA expression to screen for lncRNAs that are more in line with ceRNA conditions. Comprehensive analysis of miRNA-mRNA and miRNA-lncRNA with negative correlation between expression levels to establish a key lncRNA-miRNA-mRNA (GLUT1) ceRNA network for ESCA.

### Statistical Analysis

Unpaired samples used unpaired t-test and paired samples used paired t-test. Multi-group cell experiments use One-way ANOVA. The correlation of gene expression was evaluated using Spearman’s correlation. The threshold of *P* < 0.05 indicates the significance of correlation.

## Results

### Pan-Cancer Analysis of GLUT1 mRNA Expression in Different Databases

**Figure 1:**
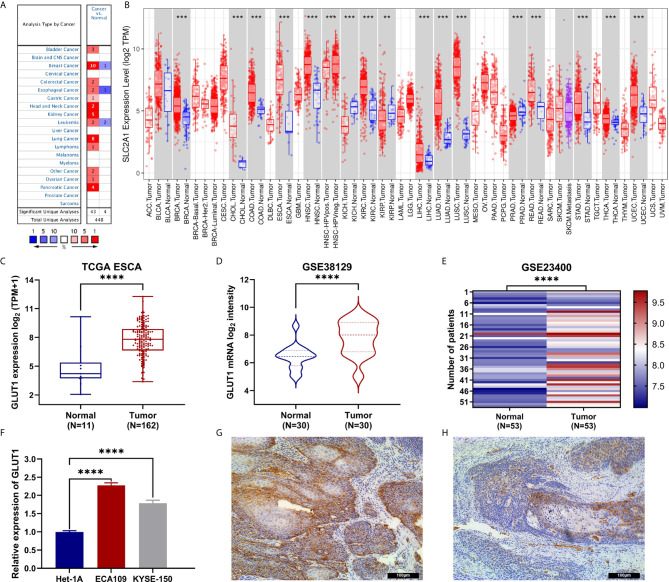
The expression of GLUT1 in esophageal carcinoma (ESCA) and pan-carcinoma. **(A)** The Oncomine database shows that GLUT1 is up-regulated in multiple tumor types. **(B)** GLUT1 expression levels in different tumor types were measured using TIMER. **(C)** TCGA cohort analysis of the expression level of GLUT1 between ESCA and normal tissues. **(D)** The GSE38129 data set was used to analyze the expression level of GLUT1 between ESCA and normal tissues. **(E)** The GSE23400 data set was used to analyze the expression level of GLUT1 between ESCA and paired normal adjacent tissues. **(F)** The expression of GLUT1 in human esophageal carcinoma ECA109 cell line, KYSE-150 cell line and human normal esophageal epithelial cells Het-1A. Immunohistochemistry assay was used to analyze the expression of GLUT1 in ESCA tissues **(G)**, in paracarcinoma tissues **(H)**. *p < 0.05; **p < 0.01; ***p < 0.001; ****p <0.0001.

To determine the difference in the expression of GLUT1 in tumors and normal tissues, the Oncomine database was used to analyze the levels of GLUT1 mRNA in tumors and normal tissues of various cancer types. This analysis showed that bladder (24, 25), breast (26–28), colorectal (29, 30), esophageal (31, 32), gastric (33), head and neck (34, 35), kidney (36–39), leukemia (40), lung (41–46), lymphoma (47), ovarian (48) and pancreatic cancer (49–52) have higher expression of GLUT1 compared with normal tissues. In addition, there are data showing that the expression is lower in breast (53), esophageal cancer (54) and leukemia tumors (55) (**Figure 1A**). **Supplementary Table 1** summarizes the details of GLUT1 expression in various cancers.

To further evaluate the expression of GLUT1 in human cancers, we used the TIMER database for analysis. The differential expression of GLUT1 in different tumors and adjacent normal tissues is shown in **Figure 1B**. Compared with adjacent normal tissues, BRCA (breast invasive carcinoma), CHOL (cholangiocarcinoma), COAD (colon adenocarcinoma), ESCA (esophageal carcinoma), HNSC (head and neck squamous cell carcinoma), KIRC (kidney renal clear cell carcinoma), KIRP (kidney renal papillary cell carcinoma), LIHC (liver hepatocellular carcinoma), LUAD (lung adenocarcinoma), LUSC (lung squamous cell carcinoma), READ (rectal adenocarcinoma), STAD (gastric adenocarcinoma), THCA (thyroid cancer) and UCEC (endometrial cancer) expression were significantly increased expression. However, compared with adjacent normal tissues, KICH (kidney chromosome) and PRAD (prostate Adenocarcinoma) have significantly lower GLUT1 expression than normal tissues.

### Transcriptional Levels of GLUT1 in Patients With ESCA

To further evaluate the expression of GLUT1 in ESCA, we used TCGA RNA sequencing data and GEO dataset for analysis and found that the level of GLUT1 mRNA was significantly increased in cancer tissues (**Figure 1C**). In unpaired or paired data sets, the expression of GLUT1 mRNA in the tumor group was significantly higher than that in normal tissues (**Figures 1D, E**). To verify the accuracy of data analysis, we also used qRT-PCR and IHC to detect the expression of GLUT1 mRNA and protein in ESCA cells. The results of qRT-PCR showed that the expression of GLUT1 mRNA in ESCA ECA109 and KYSE-150 cell lines was significantly higher than that in human normal esophageal epithelial cells Het-1A cell line (**Figure 1F**). IHC results showed that the protein level of GLUT1 in tumor tissues was significantly higher than that in adjacent normal tissues (**Figures 1G, H**). These results indicate that GLUT1 has a potential carcinogenic effect on the progression of ESCA.

### Relationship Between GLUT1 mRNA Expression and Clinicopathological Parameters in Patients with ESCA

To better understand the relevance of GLUT1 expression in cancer, we used the TCGA cohort to analyze its underlying mechanism and correlate it with certain clinical aspects. Chi-square test was performed on samples of ESCA with qualified clinical information, and it was found that the high expression of GLUT1 was significantly correlated with tissue type (*P* < 0.001) and histological grade (*P* = 0.006) (**Table 1**).

**Table 1:** Correlation of GLUT1 mRNA expression with clinicopathological features in the TCGA cohort.

Characteristic	levels	GLUT1 expression		*P*
		Low (%)	High (%)	
Age	<=60	36 (22.2%)	47 (29%)	0.116
	>60	45 (27.8%)	34 (21%)	
Gender	Female	11 (6.8%)	12 (7.4%)	1.000
	Male	70 (43.2%)	69 (42.6%)	
T stage	T1	16 (11%)	11 (7.6%)	0.085
	T2	12 (8.3%)	25 (17.2%)	
	T3	39 (26.9%)	38 (26.2%)	
	T4	3 (2.1%)	1 (0.7%)	
N stage	N0	27 (18.8%)	39 (27.1%)	0.291
	N1	35 (24.3%)	28 (19.4%)	
	N2	4 (2.8%)	5 (3.5%)	
	N3	4 (2.8%)	2 (1.4%)	
M stage	M0	58 (45%)	63 (48.8%)	0.486
	M1	5 (3.9%)	3 (2.3%)	
Pathologic stage	Stage I	8 (5.6%)	8 (5.6%)	0.064
	Stage II	26 (18.3%)	43 (30.3%)	
	Stage III	30 (21.1%)	19 (13.4%)	
	Stage IV	5 (3.5%)	3 (2.1%)	
Histological type	Adenocarcinoma	61 (37.7%)	19 (11.7%)	**<0.001**
	Squamous Cell Carcinoma	20 (12.3%)	62 (38.3%)	
Histologic grade	G1	2 (1.6%)	14 (11.1%)	**0.006**
	G2	33 (26.2%)	33 (26.2%)	
	G3	26 (20.6%)	18 (14.3%)	

Bold values indicate P < 0.05.

### Enrichment Analysis of GLUT1 Gene Co-Expression Network and PPI Analysis in ESCA

**Figure 2:**
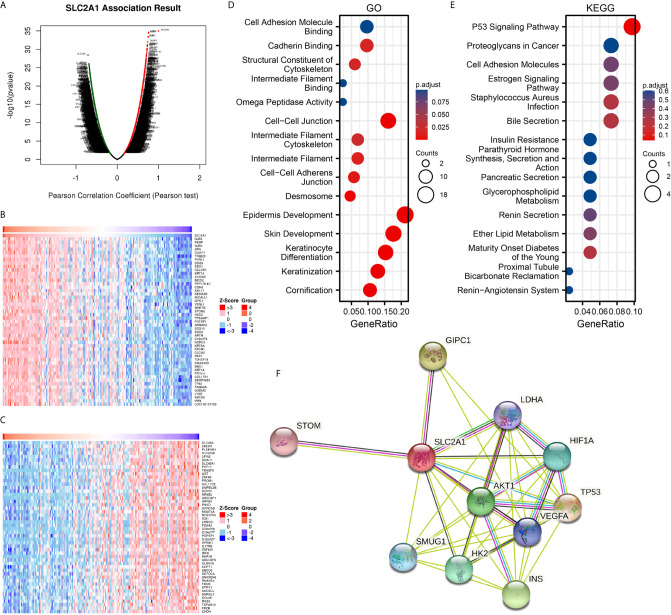
Enrichment analysis of GLUT1 functional networks in esophageal carcinoma (ESCA). **(A)** Genes highly related to GLUT1 identified in the ESCA cohort by Pearson test. **(B)** The heat map shows the top 50 genes positively related to GLUT1 in the ESCA cohort. **(C)** The heat map shows the top 50 genes negatively related to GLUT1 in the ESCA cohort. **(D)** Enrichment of gene ontology (GO) terms for genes related to GLUT1. **(E)** Enrichment of Kyoto Encyclopedia of Genes and Genomes (KEGG) terms for genes related to GLUT1. **(F)** Protein–protein interaction network of GLUT1.

To further understand the biological significance of GLUT1 in ESCA, we used the LinkedOmics database to analyze the GLUT1 co-expression in ESCA. As shown in **Figure 2A**, 4300 genes are positively correlated with GLUT1, and 6056 genes are significantly negatively correlated with GLUT1 (FDR < 0.05). The heat map shows the top 50 significant genes that are positively correlated (**Figure 2B**) and negatively correlated with GLUT1 (**Figure 2C**), respectively. See **Supplementary Table 2** for detailed descriptions of related genes.

We use R software package to perform Gene ontology (GO) and Kyoto Encyclopedia of Genes and Genomes (KEGG) enrichment analysis of GLUT1 related genes. Under the condition of p.adj < 0.1, there are 84 biological process (GO-BP), 8 cellular component (GO-CC), 5 biological process (GO-MF), and 1 KEGG. The bubble chart shows the first 15 pieces of information about GO and KEGG, including 5 pieces of BP, CC, and MF. GO function annotation shows that GLUT1 co-expression are mainly involved in structural constituent of cytoskeleton, intermediate filament binding, cell-cell adheres junction, epidermis development (**Figure 2D**). KEGG pathway analysis showed that GLUT1 co-expression are mainly related to the P53 signaling pathway (**Figure 2E**). **Supplementary Table 3** summarizes the GO and KEGG enrichment analysis details of GLUT1 co-expression.

To further understand the potential mechanism of GLUT1, the STRING database was used to study the PPI network of GLUT1. The analysis showed that GLUT1 is associated with Cellular tumor antigen p53 (TP53), PDZ domain-containing protein GIPC1 (GIPC1), Insulin (INS), RAC-alpha serine (AKT1), Hexokinase-2 (HK2), Hypoxia-inducible factor 1-alpha (HIF1A), Single-strand selective monofunctional uracil DNA glycosylase (SMUG1), Vascular endothelial growth factor A (VEGFA), Lactate dehydrogenase A (LDHA) and Erythrocyte band 7 integral membrane protein (STOM) were 0.95, 0.893, 0.873, 0.872, 0.867, 0.852, 0.831, 0.831, 0.818 and 0.817 (**Figure 2F** and **Supplementary Table 4**). TP53 plays an inhibitory role in most tumors, and GIPC1 and INS play a promoting role in some tumors. We found that the above three proteins have the highest correlation with GLUT1. These results may indicate that GLUT1 is closely related to the occurrence and development of ESCA.

### GLUT1 Expression Is Associated With Immune Signatures in ESCA

**Figure 3:**
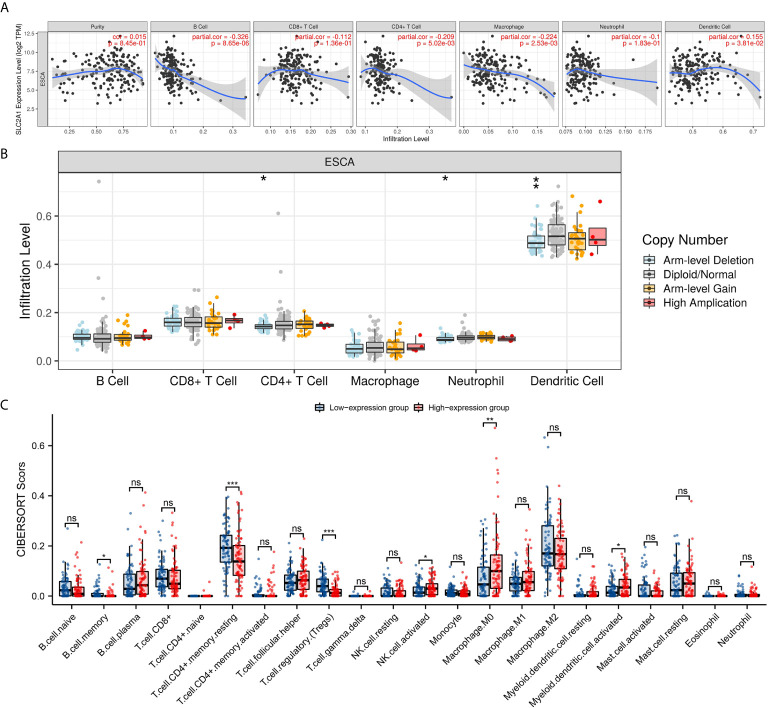
Correlations of GLUT1 expression with immune infiltration level in esophageal carcinoma (ESCA). **(A)** The expression of GLUT1 was significantly correlated with infiltrating levels of B cell, CD4+T cells, macrophages, and dendritic cells in ESCA. **(B)** GLUT1 CNV affects the infiltrating levels of CD4+T cells, neutrophils, and dendritic cells in ESCA. **(C)** The change ratio of 22 immune cell subtypes in the high and low GLUT1 expression groups in ESCA tumor samples. *p < 0.05; **p < 0.01; ***p < 0.001; ****p <0.0001. ns, not significant.

Studies have shown that tumor-infiltrating lymphocytes can be used as an independent predictor of the status and prognosis of cancer sentinel lymph nodes (56). Therefore, we used TIMER to analyze whether the expression of GLUT1 is related to the level of immune infiltration in ESCA. As shown in **Figure 3A**, GLUT1 expression showed a negative correlation with the levels of B cells (*P* = 8.65×10^-6^), CD4 + T cells (*P* = 5.02×10^-3^), Macrophages (*P* = 2.53×10^-3^) and Dendritic cells (*P* = 3.81×10^-2^). These results indicate that GLUT1 plays a key role in the immune infiltration of ESCA. In addition, it was also found that GLUT1 CNV has a significant correlation with the infiltration level of CD4 + T cells, Neutrophils and Dendritic Cells (**Figure 3B**).

To study the relationship between GLUT1 and various immune-infiltrating cells in ESCA, the TIMER tool was used to analyze the correlation between GLUT1 and immune markers of various immune cells in ESCA (**Table 2**). The results showed that the expression of GLUT1 was significantly correlated with the immune markers CD20 and CD19 of B cells in ESCA (*P* < 0.05, **Table 2**). We also analyzed a variety of T cells with different functions, such as CD8 + T cells, Tfh cells, Th1 cells, Th2 cells, Th17 cells, Treg, and exhausted T cells. The results after adjustment of tumor purity showed that the expression level of GLUT1 was significantly correlated with most of the immune markers of different T cells in ESCA. including CD8B, CD183, CD185, CD212, CD195, CD194, CD365, IL23R, CD196, and PD-1 (*P* < 0.05, **Table 2**). It indicates that GLUT1 may be involved in the T cell immune response in ESCA. We also found that the expression level of GLUT1 was significantly correlated with the immune markers nitric oxide synthase 2 (NOS2) and interferon regulatory factor 5 (IRF5) of M1 macrophage in ESCA (*P* < 0.05, **Table 2**). It indicates that GLUT1 may regulate macrophage polarization in ESCA. We also found that the expression of GLUT1 was significantly correlated with immune markers of NK cells, Neutrophil and DC in ESCA, including CD57, CD7, CD55 and CD141 (*P* < 0.05, **Table 2**). These results indicate that the expression of GLUT1 in ESCA is related to immune cell infiltration in different ways.

**Table 2:** Correlation analysis between GLUT1 and relate genes and markers of immune cells in TIMER.

Gene markers	Gene markers	rho	p	adj.p
B cell	CD19	-0.29406	6.16E-05*	4.83E-04*
	CD20	-0.32504	8.51E-06*	8.10E-05*
	CD70	0.019655	7.93E-01	8.78E-01
CD8+ T Cell	CD8A	-0.17305	2.02E-02*	5.51E-02
	CD8B	-0.25575	5.30E-04*	2.90E-03*
	CD25	-0.11128	1.37E-01	2.48E-01
Tfh	CD183	-0.35906	7.43E-07*	1.02E-05*
	CD185	-0.20494	5.78E-03*	2.07E-02*
	CD278	-0.09349	2.12E-01	3.37E-01
Th1	CD212	-0.28929	8.19E-05*	5.96E-04*
	CD191	-0.10567	1.58E-01	2.75E-01
	CD195	-0.2203	2.96E-03*	1.23E-02*
Th2	CD194	-0.3358	4.06E-06*	4.22E-05*
	CD198	-0.15153	4.23E-02*	9.99E-02
	CD365	-0.44608	3.48E-10*	9.93E-09*
Th17	CD360	-0.16464	2.72E-02*	6.98E-02
	IL23R	-0.33128	5.56E-06*	5.56E-05*
	CD196	-0.57397	3.68E-17*	5.88E-15*
Treg	FOXP3	-0.1535	3.97E-02*	9.50E-02
	CD73	-0.11494	1.24E-01	2.28E-01
	CD127	0.105172	1.60E-01	3.00E-01
T cell exhaustion	PD-1	-0.20453	5.89E-03*	2.53E-02*
	CTLA4	-0.16284	2.90E-02*	8.42E-02
	LAG3	-0.02181	7.71E-01	8.79E-01
Macrophage	CD68	0.003556	9.62E-01	9.86E-01
	CD11b	-0.11248	1.33E-01	2.60E-01
M1 Macrophage	NOS2	-0.37043	3.08E-07*	4.93E-06*
	IRF5	0.384121	1.02E-07*	1.85E-06*
M2 Macrophage	CD163	-0.12681	8.98E-02	1.94E-01
	CD206	-0.00262	9.72E-01	9.89E-01
TAM	CCL2	0.005064	9.46E-01	9.79E-01
	CD86	0.107275	1.52E-01	2.87E-01
Monocyte	CD14	-0.01487	8.43E-01	9.31E-01
	CD33	-0.08073	2.81E-01	4.45E-01
Natural killer cell	CD57	-0.26358	3.50E-04*	2.46E-03*
	KIR3DL1	-0.13801	6.47E-02	1.53E-01
	CD7	-0.25512	5.48E-04*	3.66E-03*
Neutrophil	CD16	0.046633	5.34E-01	6.82E-01
	CD55	-0.38651	8.36E-08*	1.59E-06*
Dendritic cell	CD1C	-0.0649	3.87E-01	5.51E-01
	CD141	0.545347	2.46E-15*	4.92E-14*

* and bold values indicate P < 0.05.

In addition, we divided 162 tumor samples into two groups based on GLUT1 expression, with 81 samples in the high-expression group and 81 samples in the low-expression group. We tried to analyze the differential expression of 22 immune cells between different GLUT1 expression groups to determine whether the tumor immune microenvironment is different between high GLUT1 expression level and low GLUT1 expression level in ESCA (**Figure 3C**). The results show that the expression of memory B cell, resting memory CD4 + T cell, regulatory T cell (Tregs), activated NK cell, M0 macrophage and activated myeloid dendritic cell are quite different between the high and low GLUT1 expression groups. The results showed that, compared with the low expression group, activated NK cell, M0 macrophage and activated myeloid dendritic cell increased in the high expression group of GLUT1 (*P* < 0.05), while the memory B cell, resting memory CD4 + T cell and regulatory T cell (Tregs) decreased (*P* < 0.05).

### GLUT1 Expression Is Associated With m6A RNA Methylation Regulators in ESCA

**Figure 4:**
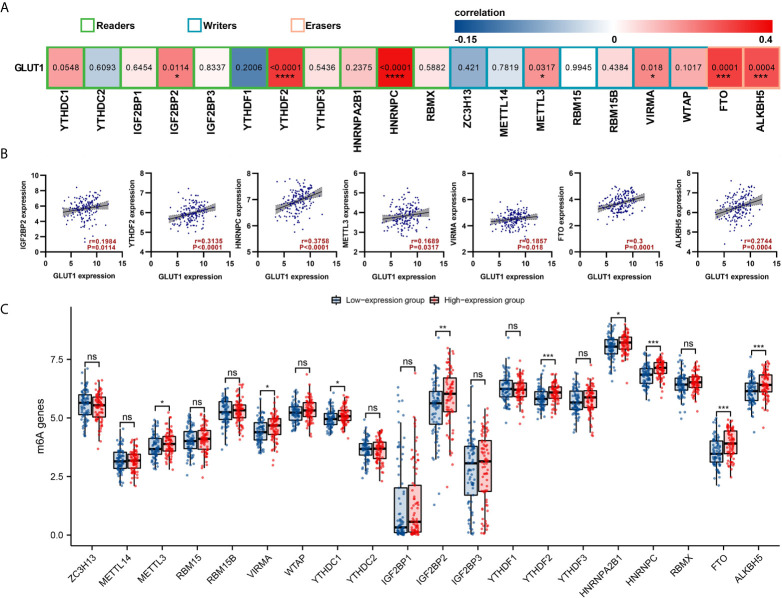
Correlations of GLUT1 expression with m6A related genes in esophageal carcinoma (ESCA). **(A)** TCGA cohort analyzed the correlation between the expression level of GLUT1 and the expression of m6A-related genes in ESCA. **(B)** Draw a scatter plot to show the correlation between GLUT1 and m6A related genes. Related m6A related genes include IGF2BP2, YTHDF2, HNRNPC, METTL3, VIRMA, FTO and ALKBH5. **(C)** The differential expression of m6A related genes in the high and low GLUT1 expression groups in ESCA tumor samples. *p < 0.05; **p < 0.01; ***p < 0.001; ****p <0.0001. ns, not significant.

The m6a modification plays an important role in the occurrence and development of ESCA. We tried to analyze whether GLUT1 expression is related to m6A modification. We analyzed the TCGA ESCA data set to study the correlation between the expression of GLUT1 and 20 m6A-related genes in ESCA (**Figure 4A**). The results showed that GLUT1 expression was significantly positively correlated with 7 m6A-related genes in ESCA, including IGF2BP2 (r = 0.1984, *P* = 0.0114), YTHDF2 (r = 0.3135, *P* < 0.0001), HNRNPC (r = 0.3758, *P* < 0.0001), METTL3 (r = 0.1689, *P* = 0.0317), VIRMA (r = 0.1857, *P* = 0.018), FTO (r = 0.3, *P* = 0.0001) and ALKBH5 (r = 0.2744, *P* = 0.0004). Draw a scatter plot to show the correlation between GLUT1 and m6A related genes (**Figure 4B**). In addition, we divided 162 tumor samples into two groups based on GLUT1 expression, with 81 samples in the high-expression group and 81 samples in the low-expression group. We tried to analyze the differential expression of 20 m6A related genes between different GLUT1 expression groups to determine whether the m6A modification is different between high GLUT1 expression level and low GLUT1 expression level in ESCA (**Figure 4C**). The results showed that, compared with the low expression group, the expression of METTL3, VIRMA, YTHDC1, IGF2BP2, YTHDF2, HNRNPA2B1, HNRNPC, FTO and ALKBH5 increased in the high expression group of GLUT1 (P < 0.05). The above results indicate that GLUT1 is closely related to m6A modification in ESCA.

### GLUT1 Related ceRNA Network Construction in ESCA

**Figure 5:**
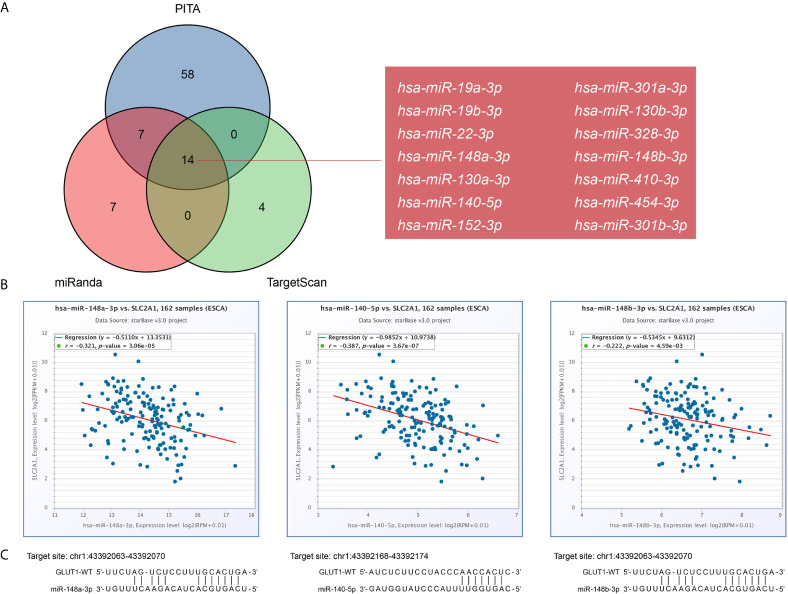
Prediction of miRNAs targeting GLUT1 in esophageal carcinoma (ESCA). **(A)** Venn graph showing the prediction results of GLUT1 targets in PITA, miRanda, and TargetScan software packages. **(B)** Use starBase software to analyze the correlation between GLUT1 and the target miRNA. Use scatter plots to show miRNA-mRNA with significant correlation. **(C)** TargetScan predicts the potential binding site of GLUT1 to the target miRNA.

There is growing evidence that the lncRNA-miRNA-mRNA ceRNA network plays a key role in a variety of human cancers, so we tried to analyze and construct a ceRNA network involving GLUT1 in ESCA. We use PITA, miRanda and TargetScan databases to analyze and predict 79, 28 and 18 GLUT1 target miRNAs, respectively. Venn diagram shows the prediction results of GLUT1 target miRNA in PITA, miRanda and TargetScan software. A total of 14 target miRNAs are common predicted by 3 databases, including hsa-miR-19a-3p, hsa-miR-19b-3p, hsa-miR-22-3p, hsa-miR-148a-3p, hsa-miR-130a-3p, hsa-miR-140-5p, hsa-miR-152-3p, hsa-miR-301a-3p, hsa-miR-130b-3p, hsa-miR-328-3p, hsa-miR-148b-3p, hsa-miR-410-3p, hsa-miR-454-3p and hsa-miR-301b-3p (**Figure 5A**). In addition, we analyze the correlation between target miRNA expression and GLUT1 expression to screen for miRNAs that are more in line with ceRNA conditions. As shown in **Figure 5B**, correlation analysis proved that there are 3 target miRNAs expression levels negatively correlated with GLUT1, namely hsa-miR-148a-3p (r = -0.321, *P* < 0.0001), hsa-miR-140-5p (r = -0.387, *P* < 0.0001) and hsa-miR-148b-3p (r = -0.222, *P* < 0.0001). TargetScan predicts the potential binding site of GLUT1 to the target miRNA (**Figure 5C**).

**Figure 6:**
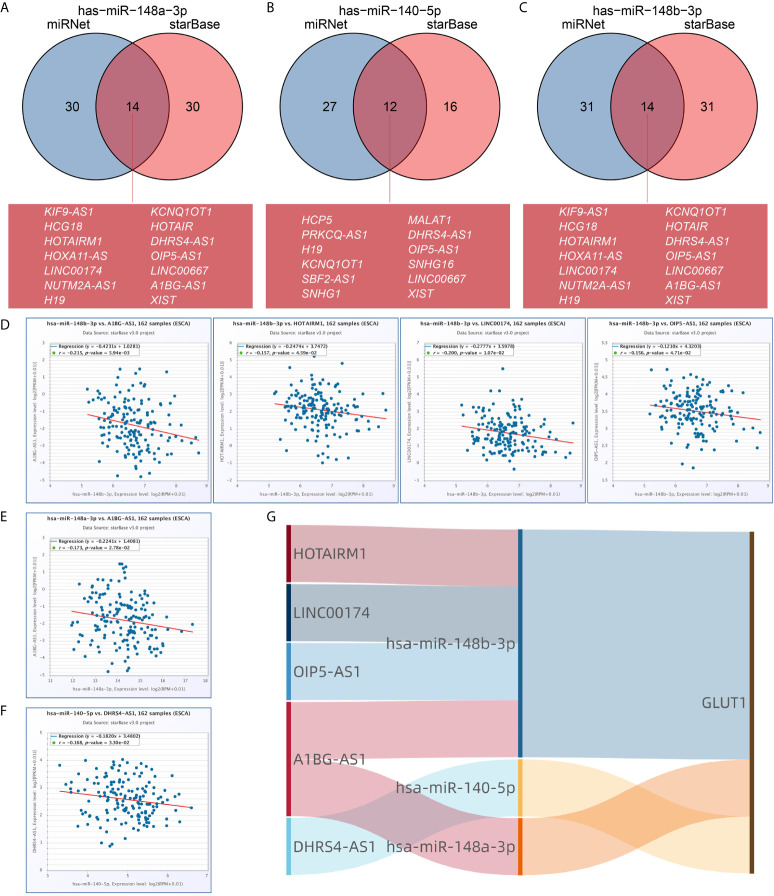
Prediction of lncRNA and ceRNA network construction in esophageal carcinoma (ESCA). The Venn diagram respectively shows the target lncRNA of has-miR-148a-3p **(A)**, has-miR-140-5p **(B)**, and has-miR-148b-3p **(C)**. Use starBase software to analyze the correlation between miRNA and the target lncRNA. Use scatter plots to show miRNA-mRNA with significant correlation. LncRNA related to has-miR-148b-3p **(D)**. LncRNA related to has-miR-148a-3p **(E)**. LncRNA related to has-miR-140-5p **(F)**. **(G)** The Sankey diagram shows the lncRNA-miRNA-mRNA (GLUT1) regulatory network in line with the ceRNA hypothesis.

We used the miRNet and starBase online database to further predict the lncRNA that may bind to the three target miRNAs (hsa-miR-148a-3p, hsa-miR-140-5p and hsa-miR-148b-3p) and display them through the Venn diagram (**Figures 6A–C**). Based on the ceRNA network hypothesis, there is a negative correlation between lncRNA and miRNA. Therefore, we used the starBase database to analyze the correlation between target lncRNA expression and miRNA in ESCA. As shown in **Figure 6D**, correlation analysis proved that there are 4 target lncRNAs expression levels that are negatively correlated with hsa-miR-148b-3p, namely HOTAIRM1, LINC00174, OIP5-AS1 and A1BG-AS1. However, only the expression level of DHRS4-AS1 was negatively correlated with hsa-miR-140-5p (**Figure 6E**), and the expression of A1BG-AS1 was negatively correlated with hsa-miR-148a-3p (**Figure 6F**). Based on the ceRNA hypothesis, there is an inverse relationship between miRNA and lncRNA or mRNA, so we can construct 6 pairs of ceRNA networks (A1BG-AS1- miR-148a-3p-GLUT1, HOTAIRM1-miR-148b-3p-GLUT1, LINC00174-miR-148b-3p-GLUT1, OIP5-AS1-miR-148b-3p-GLUT1, A1BG-AS1-miR-148b-3p-GLUT1 and DHRS4-AS1-miR-140-5p-GLUT1) based on the correlation analysis results (**Figure 6G**).

## Discussion

GLUTs are mainly distributed in the cell membrane area and can mediate glucose transport across the membrane. The up-regulation of GLUTs mainly exists in most tumor cells. It is a key factor for the uptake of glucose by tumor cells and may also be one of the causes of early carcinogenesis (57). Among the known diseases in humans, mutations in the GLUT1 gene affect the normal uptake of glucose by cells, which in turn triggers a series of diseases such as brain atrophy and developmental delay. But in cancer, tumor cells need to take in a lot of glucose to maintain the malignant proliferation of cells, so the expression of GLUT1 will eventually affect the development of cancer (11). Studies have shown that GLUT1 is highly expressed in a variety of cancers, and the expression level is closely related to clinical pathological characteristics such as tumor stage and tumor grade (4–8). In this study, we verified the differential expression of GLUT1 in tumors through experiments and bioinformatics analysis.

Analysis of the Oncomine database found that GLUT1 was highly expressed in 12 cancers, and the TCGA cohort showed that GLUT1 was highly expressed in 13 cancers, which is consistent with previous studies. Through the analysis of GEO and TCGA ESCA cohort, it was found that compared with normal tissues, the expression of GLUT1 in ESCA samples was significantly increased (*P* < 0.05). We also detected the expression of GLUT1 in ESCA samples and normal tissue samples by qRT-PCR and IHC, and the analysis results were the same as above. Kato et al. (58) found that the expression of GLUT1 in ESCA is closely related to the tumor status, metastatic status, lymph node status and pathological stage of patients. This is different from the results of our analysis using TCGA ESCA cohort, which may be due to the large difference in sample size. However, many researchers have found that ESCA patients with high GLUT1 expression have a worse prognosis than those with low GLUT1 expression (58–60). In summary, GLUT1 can be used as a potential diagnostic and prognostic marker for ESCA.

At present, the research on the function and mechanism of GLUT1 in tumors mainly focuses on the glycolysis process of tumor cells. Zheng et al. (61) found that Circ_0058063 up-regulates GLUT1 expression and promotes glucose uptake in ESCC, thereby promoting cell proliferation. However, no study on the functional enrichment analysis of GLUT1 co-expression in ESCA has been reported. In this study, we used the LinkedOmics database to analyze the GLUT1 co-expression in ESCA. Through the GO and KEGG functional enrichment analysis of 100 genes related to GLUT1, it is found that the GLUT1 co-expression is mainly related to the structural constituent of cytoskeleton, intermediate filament binding, cell-cell adheres junction, and epidermis development. KEGG pathway analysis showed that the GLUT1 co-expression was mainly related to the P53 signaling pathway. These functions and pathways are all related to the occurrence and development of ESCA. PPI analysis found that GLUT1 has the strongest correlation with TP53, GIPC1 and INS, and these three proteins all play important roles in the proliferation of tumor. The above results suggest that GLUT1 may not only participate in glycolysis in ESCA, but may also have multiple biological functions.

To explore the relationship between GLUT1 and immune infiltration in ESCA, we used the TIMER database to reveal the relationship between GLUT1 expression and the level of immune infiltration in ESCA. We found that GLUT1 expression is significantly correlated with B cells, CD4 + T cells, macrophages, and dendritic cells. In addition, it was also found that GLUT1 CNV was significantly correlated with the infiltration level of CD4 + T cells, neutrophils, and dendritic cells. Moreover, the correlation between GLUT1 expression and immune cell marker genes suggests that GLUT1 plays an important role in regulating ESCA tumor immunity. TIMER database analysis found that GLUT1 expression has a significant correlation with the gene markers of B cells (CD19, CD20), CD8 + T Cell (CD8B), Tfh (CD183, CD185), Th1 (CD212, CD195), Th2 (CD194, CD365), Th17 (IL23R, CD196), T cell exhaustion (PD-1), M1 macrophage (NOS2, IRF5), NK cell (CD57), neutrophil (CD55), and DC (CD141). We also found that the level of NK cell, M0 macrophage and activated myeloid dendritic cell increased in the GLUT1 high expression group, while the levels of memory B cell, resting memory CD4 + T cell and regulatory T cell decreased. We believe that GLUT1 can affect the immune infiltration of ESCA by regulating memory B cell. After activating a human’s primary immunity, B cells will proliferate in large numbers, most of which will differentiate into effector B cells, and the rest will differentiate into memory B cells. Memory B cells exist in the human body for a long time and can play a long-term role. Ledesma et al. (62) found that the number of memory B cells in immune responders increased greatly, and they played a role in inhibiting tumor cell proliferation and metastasis. We can infer that the overexpression of GLUT1 inhibits the immune response and infiltration of memory B cells. We believe that excessive GLUT1 in patients with ESCA will trigger an anti-tumor immune response. These findings suggest that GLUT1 plays an important role in the regulation and recruitment of immune infiltrating cells in ESCA. However, controlled trials and clinical trials are needed to explain the relationship more accurately between GLUT1 and memory B cells *in vivo*.

M6A methylation is the most common form of mRNA modification in eukaryotes, and it plays a vital role in promoting tumor proliferation and migration. Chen et al. (11) found that the key m6A gene METTL3 can enhance the stability of GLUT1 mRNA, increase the glucose uptake and lactate production of colorectal cancer cells, and promote the development of colorectal cancer. Huang et al. (63) found that IGF2BP2 directly binds to GLUT1 mRNA and stabilizes GLUT1 mRNA, thereby promoting aerobic glycolysis and proliferation of pancreatic ductal adenocarcinoma cells. In this study, we tried to analyze whether GLUT1 expression is related to m6A modification in ESCA. We found that GLUT1 expression was significantly correlated with IGF2BP2, YTHDF2, HNRNPC, METTL3, VIRMA, FTO and ALKBH5. We also found that the levels of METTL3, VIRMA, YTHDC1, IGF2BP2, YTHDF2, HNRNPA2B1, HNRNPC, FTO and ALKBH5 increased in the GLUT1 high expression group. We believe that the GLUT1 gene may be modified by m6A to enhance mRNA stability, which in turn enhances the glycolysis and proliferation of ESCA.

The crosstalk between ceRNA is achieved by long non-coding RNA (lncRNA) or circular RNA (circRNA) competitively binding miRNA to affect mRNA expression. lncRNA and circRNA play the role of miRNA sponge, reducing the abundance of miRNA in the body, thereby reducing the inhibitory effect of miRNA on downstream target genes. Li et al. (64) found that lncRNA RAD51-AS1 can inhibit the miR-29b/c-3p/NDRG2 signal axis and the expression of hexokinase 2 and GLUT1, thereby inhibiting the progression of colorectal cancer. Chen et al. (12) found that circRNA_100290 can be used as ceRNA to eliminate the inhibitory effect of mir-378a on GLUT1, thereby promoting glycolysis and proliferation of oral squamous cell carcinoma. In this study, we first predicted some upstream miRNAs of GLUT1. The 3 databases jointly predicted 14 potential upstream miRNAs, but the expression of only 3 miRNAs (hsa-miR-148a-3p, hsa-miR-140-5p, hsa-miR-148b-3p) was significantly negatively correlated with GLUT1 in ESCA. Mari et al. (65) reported that overexpression of miR-148a-3p in ESCA can enhance tumor immune response. Chen et al. (66) found that miR-140-5p is under-expressed in ESCA, and it may regulate the cell invasion of ESCA by regulating the expression of Slug. Then, we further predicted the upstream lncRNA of these key miRNAs. Through correlation analysis, only 5 lncRNAs (HOTAIRM1, LINC00174, OIP5-AS1, A1BG-AS1, DHRS4-AS1) can be defined as key lncRNAs. HOTAIRM1 is up-regulated in glioblastoma and promotes tumor cell migration and invasion (67). LINC00174 targets miR-4500 in laryngeal papilloma to inhibit BZW2 and promote tumor cell proliferation (68). OIP5-AS1 promotes the invasion and migration of ovarian cancer cells (69). These reports further hint at the feasibility of our analysis. Of course, although the ceRNA network of GLUT1 was obtained through bioinformatics analysis, more experiments are needed to confirm our prediction.

In summary, this is the first comprehensive analysis of the relationship between GLUT1 expression and tumor immune infiltration, m6A modification, and ceRNA network in ESCA. The GLUT1 gene may be modified by m6A to enhance the stability of its mRNA, thereby enhancing the effect of promoting glycolysis and cell proliferation in ESCA. The expression of GLUT1 is related to a variety of immune cells and may affect ESCA tumor immunity by inhibiting the infiltration of memory B cells. The construction of ceRNA network of GLUT1 indicates that GLUT1 may participate in a variety of molecular regulatory mechanisms in ESCA. GLUT1 can be used as an effective biomarker for the diagnosis and treatment of ESCA.

## Data Availability Statement

The original contributions presented in the study are included in the article/**Supplementary Material**. Further inquiries can be directed to the corresponding author.

## Ethics Statement

The studies involving human participants were reviewed and approved by Ethics Committee of Taihe Hospital Affiliated of Hubei University of Medicine. Written informed consent for participation was not required for this study in accordance with the national legislation and the institutional requirements.

## Author Contributions

X-SL, YG, and L-BW conceived the project and wrote the manuscript. X-SL, H-BW, PY, YJ, S-BG, Y-LW, and X-QC participated in data analysis. L-MZ, J-WY, X-YK, and X-YL participated in discussion and language editing. Z-JP reviewed the manuscript. All authors contributed to the article and approved the submitted version.

## Funding

This work was supported by the Hubei province’s Outstanding Medical Academic Leader program, the Foundation for Innovative Research Team of Hubei Provincial Department of Education T2020025, the Hubei Provincial Department of Science and Technology Innovation Group Program (grant no. 2019CFA034), Free-exploring Foundation of Hubei University of Medicine (grant no. FDFR201903), Open Project of Hubei Key Laboratory of Embryonic Stem Cell Research (grant no.2020ESOF009), and the Key Discipline Project of Hubei University of Medicine.

## Conflict of Interest

The authors declare that the research was conducted in the absence of any commercial or financial relationships that could be construed as a potential conflict of interest.

## Supplementary Material

Supplementary Table 1 GLUT1 expression in cancerous versus normal tissue in ONCOMINE.

Supplementary Table 2 GLUT1 co-expressed genes.

Supplementary Table 3 The GO and KEGG enrichment analysis of GLUT1 related genes.

Supplementary Table 4 The PPI network of GLUT1.
