# Supplementary material for: Comprehensive Analysis of GLUT1 Immune Infiltrates and ceRNA Network in Human Esophageal Carcinoma
Source: Front Oncol. 2021 May 28;11:665388. doi: 10.3389/fonc.2021.665388 (PMC8195627; doi:10.3389/fonc.2021.665388)
Supplement: Supplementary Table 1 — GLUT1 expression in cancerous versus normal tissue in ONCOMINE. [file Table_1.docx]

Supplementary Table 1. GLUT1 expression in cancerous versus normal tissue in ONCOMINE.

| **Cancer Site** | **Cancer Type** | **P Value** | **t‑Test** | **Fold Change** | **Reference (PMID)** |
| --- | --- | --- | --- | --- | --- |
| Bladder | Infiltrating Bladder Urothelial Carcinoma | 1.85E-12 | 7.682 | 2.514 | 16432078 |
|  | Superficial Bladder Cancer | 1.20E-14 | 10.564 | 3.977 | 16432078 |
|  | Infiltrating Bladder Urothelial Carcinoma | 7.32E-6 | 5.530 | 2.274 | 15173019 |
| Breast | Invasive Ductal Breast Carcinoma | 1.03E-11 | 9.276 | 2.800 | 15034139 |
|  | Lobular Breast Carcinoma | 6.62E-6 | 5.631 | 2.075 | 15034139 |
|  | Intraductal Cribriform Breast Adenocarcinoma | 2.50E-9 | 11.263 | 2.172 | TCGA Breast |
|  | Male Breast Carcinoma | 2.48E-5 | 11.010 | 3.575 | TCGA Breast |
|  | Invasive Ductal Breast Carcinoma | 4.19E-27 | 13.974 | 2.557 | TCGA Breast |
|  | Invasive Breast Carcinoma | 8.98E-15 | 8.629 | 2.251 | TCGA Breast |
|  | Ductal Breast Carcinoma | 1.07E-6 | 6.053 | 2.340 | 16473279 |
|  | Medullary Breast Carcinoma | 4.82E-10 | 8.059 | 2.728 | 22522925 |
|  | Mucinous Breast Carcinoma | 6.44E-13 | 8.635 | 2.100 | 22522925 |
|  | Invasive Breast Carcinoma | 1.49E-5 | 5.207 | 2.317 | 22522925 |
|  | Invasive Breast Carcinoma | 4.56E-17 | -11.709 | -3.780 | 18438415 |
| Colorectal | Colon Adenocarcinoma | 1.22E-13 | 8.619 | 2.107 | 17640062 |
|  | Colorectal Carcinoma | 1.64E-9 | 7.030 | 2.372 | 20957034 |
| Esophageal | Esophageal Squamous Cell Carcinoma | 6.33E-6 | 5.692 | 2.424 | 20955586 |
|  | Esophageal Squamous Cell Carcinoma | 6.18E-13 | 8.162 | 2.134 | 21385931 |
|  | Barrett's Esophagus | 1.57E-10 | -9.953 | -3.261 | 21152079 |
| Gastric | Gastric Intestinal Type Adenocarcinoma | 3.11E-8 | 5.988 | 2.265 | 12925757 |
| Head-Neck | Tongue Squamous Cell Carcinoma | 8.20E-8 | 6.663 | 4.612 | 18254958 |
|  | Tongue Carcinoma | 8.11E-6 | 5.060 | 2.154 | 17510386 |
| Kidney | Non-Hereditary Clear Cell Renal Cell Carcinoma | 9.35E-13 | 11.104 | 5.519 | 19470766 |
|  | Hereditary Clear Cell Renal Cell Carcinoma | 7.36E-13 | 13.447 | 5.810 | 19470766 |
|  | Clear Cell Renal Cell Carcinoma | 4.00E-6 | 6.336 | 2.224 | 17699851 |
|  | Clear Cell Renal Cell Carcinoma | 1.63E-12 | 11.171 | 2.912 | 16115910 |
|  | Clear Cell Renal Cell Carcinoma | 2.27E-5 | 6.863 | 2.890 | 19445733 |
| Leukemia | B-Cell Acute Lymphoblastic Leukemia | 1.54E-11 | 11.384 | 3.218 | 17410184 |
|  | Acute Myeloid Leukemia | 1.35E-6 | 5.913 | 2.388 | 17410184 |
|  | Pro-B Acute Lymphoblastic Leukemia | 4.74E-21 | -11.337 | -2.608 | 20406941 |
|  | T-Cell Acute Lymphoblastic Leukemia | 4.15E-23 | -12.601 | -2.714 | 20406941 |
| Lung | Lung Adenocarcinoma | 1.12E-10 | 8.781 | 3.160 | 17540040 |
|  | Lung Adenocarcinoma | 2.39E-23 | 14.833 | 5.153 | 22613842 |
|  | Squamous Cell Lung Carcinoma | 1.92E-23 | 26.718 | 22.600 | 20421987 |
|  | Lung Adenocarcinoma | 2.68E-14 | 10.393 | 4.801 | 20421987 |
|  | Large Cell Lung Carcinoma | 2.44E-6 | 6.267 | 3.436 | 20421987 |
|  | Lung Adenocarcinoma | 5.86E-21 | 13.641 | 2.843 | 22080568 |
|  | Lung Adenocarcinoma | 5.99E-8 | 9.582 | 3.190 | 12118244 |
|  | Lung Adenocarcinoma | 2.15E-16 | 10.850 | 2.086 | 18297132 |
| Lymphoma | Anaplastic Large Cell Lymphoma, ALK-Positive | 1.13E-5 | 14.945 | 6.087 | 19657361 |
| Ovarian | Ovarian Serous Adenocarcinoma | 7.49E-10 | 11.221 | 7.952 | 19486012 |
| Pancreas | Pancreatic Carcinoma | 6.24E-19 | 14.773 | 8.453 | 19732725 |
|  | Pancreatic Adenocarcinoma | 7.90E-6 | 7.315 | 13.073 | 12651607 |
|  | Pancreatic Adenocarcinoma | 6.78E-6 | 7.336 | 3.587 | 12750293 |
|  | Pancreatic Ductal Adenocarcinoma | 2.48E-8 | 6.583 | 2.767 | 19260470 |
